# Health Risk Assessment of Potentially Toxic Element Uptake by Lotus (*Nelumbo nucifera*) in Floating Lake Gardens

**DOI:** 10.3390/toxics13040306

**Published:** 2025-04-13

**Authors:** Mohssen Elbagory, Farahat S. Moghanm, Ibrahim Mohamed, Sahar El-Nahrawy, Alaa El-Dein Omara, Madhumita Goala, Pankaj Kumar, Boro Mioč, Željko Andabaka, Ivan Širić

**Affiliations:** 1 Health Specialties, Basic Sciences and Applications Unit, Applied College, King Khalid University, Mohayil Asir Abha 61421, Saudi Arabia; mhmohammad@kku.edu.sa; 2 Soil and Water Department, Faculty of Agriculture, Kafrelsheikh University, Kafr El-Sheikh 33516, Egypt; fsaadr@yahoo.ca; 3 Department of Soil and Water Sciences, Faculty of Agriculture, Benha University, Moshtohor, Toukh 13736, Egypt; ibrahim.ali@fagr.bu.edu.eg; 4 Soil Microbiology Research Department, Soils, Water, and Environment Research Institute (SWERI), Agriculture Research Center (ARC), Giza 12112, Egypt; sahar.elnahrawy@yahoo.com (S.E.-N.); alaa.omara@yahoo.com (A.E.-D.O.); 5 Department of Environmental Science, Graphic Era (Deemed to be University), Dehradun 248002, India; 6 School of Environmental Studies, Maa Shakumbhari University, Punwarka, Saharanpur 247120, India; 7 Research and Development Division, Society for AgroEnvironmental Sustainability, Dehradun 248007, India; 8 Faculty of Agriculture, University of Zagreb, Svetosimunska 25, 10000 Zagreb, Croatia; bmioc@agr.hr (B.M.);

**Keywords:** bioaccumulation, floating lake gardens, health risk assessment, lotus cultivation, toxic element contamination

## Abstract

The present study investigated the uptake and health risks of potentially toxic elements (PTEs) by lotus (*Nelumbo nucifera*) cultivated in floating lake gardens of Dal Lake, Srinagar, India. Rapid urbanization and anthropogenic activities have led to PTE contamination in the lake, raising concerns about food safety and ecological sustainability. The objectives were to evaluate the physicochemical properties of water and sediment and to quantify PTEs (Cd, Cu, Cr, Co, Fe, Mn, Ni, and Zn) accumulation in different tissues of *N. nucifera* with associated health risks. A systematic sampling approach was adopted across four zones of the lake, collecting water, sediment, and plant tissues (August to October 2024). The results showed significant PTE contamination, with sediment showing high concentrations of Fe (1610.51 mg/kg), Mn (31.48 mg/kg), and Cr (29.72 mg/kg). Bioaccumulation factors indicated preferential PTE accumulation in roots, with Fe exhibiting the highest uptake (95.11). Translocation factors were low for most PTEs, suggesting limited mobility to edible parts. Health risk assessment indicated that Cr posed the highest non-carcinogenic risk (HRI = 1.8000 in rhizomes). The cumulative target hazard quotient (THQ) remained below 1, suggesting no immediate risk, but long-term exposure warrants concern. The study provided valuable information on the necessity of continuous monitoring and pollution mitigation strategies to ensure the food safety of floating lake garden-derived crops.

## 1. Introduction

The lotus (*Nelumbo nucifera*), commonly known as the sacred lotus, is an aquatic plant with deep evolutionary and ecological significance [1]. Fossil records suggest that *N. nucifera* has existed for over 135 million years, with its lineage dating back to the Cretaceous period [2]. Its distribution is largely affected by precipitation and availability of water bodies [3,4]. The species has developed specialized adaptations, such as hydrophobic leaves, thermoregulation in flowers, and seed dormancy, which allow it to thrive in aquatic environments [5]. *N. nucifera* plays an important role in stabilizing wetland ecosystems by improving water quality, controlling algal blooms, and providing habitat for various aquatic organisms [6,7]. Apart from its ecological importance, *N. nucifera* has been widely used in traditional medicine, culinary practices, and religious ceremonies [8]. The rhizomes, seeds, leaves, and flowers of *N. nucifera* contain bioactive compounds such as alkaloids, flavonoids, and polyphenols, which exhibit antioxidant, anti-inflammatory, and antimicrobial properties [9]. *N. nucifera* is valued in Asian cultures for its nutritional [10] and medicinal properties, used in Ayurveda and traditional Chinese medicine for various ailments, and symbolizes purity and enlightenment in Hinduism and Buddhism [11]. Thus, due to its wide ecological, medicinal, and cultural applications, *N. nucifera* is one of the significant aquatic plants in both natural and human-influenced landscapes [12].

*N. nucifera* horticulture practice is widespread across Asia, particularly in China, India, Japan, and Southeast Asian countries, where it is grown for food, medicine, and ornamental purposes [13,14]. China is the leading producer, cultivating *N. nucifera* on large-scale aquatic farms, particularly in provinces like Hubei and Jiangxi, where the plant is used for seed and rhizome production [15]. In India, *N. nucifera* is grown in the water bodies of Uttar Pradesh, Bihar, Kashmir, and Tamil Nadu states [16]. The edible parts of *N. nucifera*, especially the rhizomes (locally called *Nadru* in Kashmir) and seeds (*Makhana*), contribute significantly to local economies [17]. The growing market for *N. nucifera* products is related to herbal teas, essential oils, and pharmaceuticals [18], alongside its cultivation for religious, aesthetic, and agro-tourism purposes [19]. Therefore, the increasing recognition of *N. nucifera* as a high-value crop has encouraged research into optimized cultivation methods, value-added processing, and market expansion to enhance its commercial viability.

Floating lake gardens are a unique agricultural system practiced in various parts of the world that provides a sustainable means of cultivating aquatic crops like *N. nucifera* [20]. These gardens are prevalent in regions with extensive water bodies such as Dal Lake in Kashmir, Inle Lake in Myanmar, and parts of Bangladesh [21,22]. Farmers construct floating beds using organic materials such as decomposed plant matter, allowing crops to grow on water surfaces. This method optimizes land use, provides livelihood opportunities, and ensures year-round cultivation. In Dal Lake, floating gardens (*Rad*) serve as an essential resource for local farmers who grow *N. nucifera* alongside vegetables and other aquatic crops [23]. The system also enhances nutrient recycling in lakes, contributing to ecological balance. However, increasing anthropogenic activities have led to severe water quality degradation in many lakes, including Dal Lake [24]. Unregulated sewage discharge, agricultural runoff, and urban expansion have introduced high levels of organic pollutants, potentially toxic elements (PTEs), and excess nutrients, leading to eutrophication and contamination of aquatic ecosystems [25,26]. Water quality assessments of Dal Lake have revealed elevated concentrations of biochemical oxygen demand (BOD), chemical oxygen demand (COD), and potentially toxic elements (PTEs) such as cadmium (Cd), lead (Pb), and chromium (Cr) [27,28]. These pollutants pose serious threats to aquatic biodiversity, disrupt ecosystem functions, and jeopardize the safety of crops grown in floating lake gardens [29].

The ability of aquatic plants to absorb and accumulate PTEs raises concerns about the safety of edible crops cultivated in polluted water bodies [30]. *N. nucifera*, with its extensive root system, can absorb and store PTEs from sediments and water, leading to bioaccumulation in rhizomes, leaves, and seeds [31]. Studies have reported high concentrations of PTEs such as Cd, Pb, nickel (Ni), and arsenic (As) in *N. nucifera* tissues when grown in contaminated water [32]. The translocation of these PTE from roots to edible parts increases potential health risks for consumers. PTE accumulation in food crops is associated with several toxicological effects, including neurotoxicity, kidney damage, and carcinogenicity [33]. Chronic exposure to Cd and Pb through dietary intake can result in bioaccumulation in human tissues, leading to oxidative stress, bone demineralization, and cardiovascular diseases [34]. The target hazard quotient (THQ) and hazard risk index (HRI) are commonly used to evaluate the potential health risks associated with consuming PTE-contaminated food [35]. Elevated THQ values indicate a significant non-carcinogenic health risk, particularly for populations that consume *N. nucifera* rhizomes as a staple [36].

Despite increasing concerns about heavy metal contamination in aquatic environments, limited research has examined the bioaccumulation of PTEs in *N. nucifera*. Previous studies have reported PTE in various macrophytes, yet there is insufficient data on the extent of PTE uptake by *N. nucifera*, particularly in highly polluted freshwater ecosystems, i.e., Dal Lake. The contamination of water and sediment by anthropogenic activities raises concerns regarding metal accumulation in edible plant parts, which may pose health risks to consumers [37]. However, no study has specifically assessed the bioaccumulation and health risks associated with *N. nucifera* growing in the floating lake gardens of Dal Lake.

Thus, this study hypothesizes that *N. nucifera* accumulates PTEs from contaminated water and sediments, leading to potential health risks. The objectives of this study were: (1) to evaluate the physicochemical properties of water and sediment, as well as the bioaccumulation and translocation patterns of PTEs in different parts of *N. nucifera*; and (2) to assess the health risks associated with the consumption of *N. nucifera* rhizomes and seeds. The findings of this research provide valuable information for metal contamination in edible aquatic plants and contribute to environmental monitoring efforts, risk assessment frameworks, and potential remediation strategies for contaminated aquatic ecosystems.

## 2. Materials and Methods

### 2.1. Study Area

**Figure 1:**
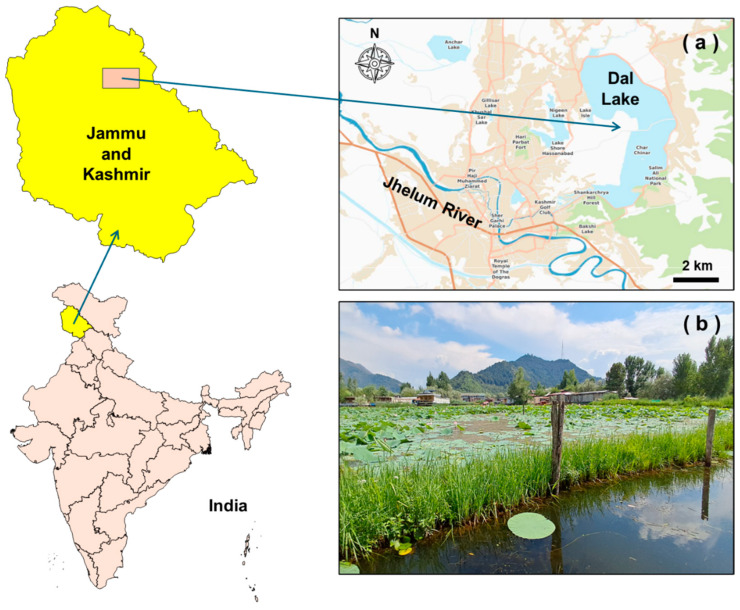
(**a**) Map of the Dal Lake located in Jammu and Kashmir state of India, and (**b**) floating lake gardens with *N. nucifera* (map created using Bhuvan 2D and Power-user).

This study was conducted in Dal Lake, Srinagar, Kashmir (34.1106° N, 74.8683° E), a freshwater lake renowned for its scenic beauty and cultural significance (Figure 1). The lake, historically fed by the Jhelum River, has transformed into an urban water body due to increasing tourism and anthropogenic activities. It has a shoreline of approximately 15.5 km and covers an area of 18 km^2^, with an average depth of 1.5 m and a maximum depth of 6 m. The lake is divided into four basins: Hazratbal, Bod Dal, Gagribal, and Nigeen. The present study was conducted between August and October 2024, coinciding with the growth and harvest period of *N. nucifera*, locally known as *Nadru*. *N. nucifera* cultivation is a traditional practice in the floating gardens (Rad in the local language) of Dal Lake, which serve as an important economic resource for local farmers. These gardens are established in shallow shore areas (1–2 m depth), where organic content-rich sediments provide suitable conditions for *N. nucifera* growth. The plant is cultivated for seeds (*Makhana*), rhizomes, and flowers, which hold nutritional, medicinal, and religious importance. Previously, the plant was genetically identified by Mehraj et al. [38] using the DNA barcoding method (MatK primer).

### 2.2. Sampling Design and Sample Collection

A comprehensive sampling approach was employed to assess the uptake of potentially toxic elements in *Nelumbo nucifera* in Dal Lake, Srinagar. Samples were collected over three phases during the *N. nucifera* growth and harvest period, specifically from August to October 2024. The study area was divided into four distinct spatial zones—Hazratbal, Bod Dal, Gagribal, and Nigeen. In each zone, three samples were collected per month, resulting in a total of 12 samples per month. Over the three-month study period, 36 samples were collected separately for water, sediment, and plant tissues. In this, water samples were collected in 5 L capacity PVC containers, following standard procedures. These containers were pre-rinsed with distilled water before sample collection. Sediment samples were collected using a Van Veen grab sampler at a bottom depth of 5–10 cm [39]. The samples were transferred into pre-cleaned polyethylene bags, stored on ice, and transported to the laboratory for analysis. On the other hand, plant samples, including *N. nucifera* rhizomes, roots, petioles, leaves, and seeds, were carefully harvested by cutting using a sharp knife and stored in sterile zip-lock polybags to prevent microbial degradation and cross-contamination. All collected samples were transported under controlled conditions to the laboratory for further physicochemical and elemental analysis.

### 2.3. Analytical Methods

Water and sediment quality parameters were analyzed to assess the physicochemical characteristics of Dal Lake. The pH of water and sediment samples was measured using a digital pH meter to determine the acidity or alkalinity. Electrical conductivity (EC) and total dissolved solids (TDS) were analyzed using a multimeter. Dissolved organic matter (DOM) was quantified using a UV-Vis spectrophotometric method [40]. Biochemical oxygen demand (BOD_5_) was determined using the Winkler method. Chemical oxygen demand (COD) was analyzed by the open reflux method, which estimates the oxidizable organic and inorganic matter in water. Total nitrogen (TN) was quantified using the Kjeldahl method [41]. Total phosphorus (TP) was estimated using a spectrophotometric method. PTE concentrations (Cd, Cu, Cr, Co, Fe, Mn, Ni, and Zn) in water and *N. nucifera* tissue were analyzed using Inductively Coupled Plasma Optical Emission Spectroscopy (ICP-OES). Water samples were acidified with nitric acid (HNO_3_) and subjected to microwave digestion before ICP-OES analysis. Sediment and plant samples were oven-dried, ground into a fine powder, and digested using a mixture of HNO_3_ and hydrogen peroxide (H_2_O_2_). The resulting digests from three matrices were filtered, diluted to 50 mL using 2% HNO_3_, and analyzed using ICP-OES for quantification of PTE concentrations [42]. Method validation was performed by assessing recovery rates using Certified Reference Materials (CRM), with analytical accuracy within an acceptable range of 96–102%. The Limit of Detection (LOD) and Limit of Quantification (LOQ) were determined for each PTE using signal-to-noise ratios of 3:1 and 10:1, respectively.

### 2.4. Data Analysis

The bioconcentration factor (BCF) and translocation factor (TF) were calculated to assess the uptake and internal mobility of elements in *N. nucifera*. The BCF was determined as the ratio of PTE concentration in plant tissues to that in water. The TF was calculated as the ratio of PTE concentration in the aerial parts to that in the roots.

(1) BAF=PTEplant/PTEwater

(2) TF=PTEaerial/PTEroot

To evaluate potential health risks, dietary intake of metal (DIM), hazard risk index (HRI), and target hazard quotient (THQ) were computed for edible plant tissues, particularly rhizomes, using established risk assessment models [35]. In this, Equations (3) and (4) were used to compute HRI and DIM values, as given below:

(3) $$HRI=\frac{DIM}{RfD}$$

(4) $$DIM=SL\times \frac{PTEc}{Bw}$$

where RfD, SL, PTEc, and Bw represent oral reference dose, serving of *N. nucifera* rhizome and seeds (dried weight), PTE concentration, and body weight of consumer (70 kg), respectively. Additionally, THQ [43] was used to evaluate the health risk of PTE accumulation in saffron as per the following model (Equation (5)):

(5) $$THQ=10^{-3}\times \frac{Ef\times Ed\times Ir\times PTEc}{Bw\times Cp\times RfD}$$

where 10^−3^ is the conversion factor, Ef is the exposure frequency, Ed is the exposure duration (365 days), Ir represents the saffron ingestion rate (0.30 kg/day), PTEc is the PTE concentration in the *N. nucifera* sample (mg/kg), Bw corresponds to the average body weight (70 kg), Cp is the consumption period (25550 days), and Rd is the reference dose in suggested by USEPA [44] terms of mg/kg/day (Cd: 1.0 × 10^−3^; Cu: 4.0 × 10^−2^; Cr: 5.0 × 10^−3^; Co: 2.0 × 10^−2^, Fe: 7.0 × 10^−1^; Mn: 1.4 × 10^−2^, Ni: 2.0 × 10^−2^, and Zn: 3.0 × 10^−1^). Further, the combined toxicity of PTE intake from *N. nucifera* was calculated as per Equation (6):

(6) ∑THQ=THQ(Cd+Cu+Cr+Co+Fe+Mn+Ni+Zn)

To determine the contribution of each PTE in *N. nucifera* uptake, the Accumulation Nutrient Elements (ANEs) method was applied [45]. The ANEs model quantifies the uptake of elements by *N. nucifera* by assessing their accumulation in plant tissues. This model helps determine the contribution of each element to overall uptake, providing insights into bioaccumulation patterns. The percentage contribution of each PTE was computed using the following model (Equation (7)):

(7) $$Y=\sum_{i=1}^{i}\left(Z\;:z\right)$$

where *Y* represents the total accumulation of participating PTE in mmol_c_/kg. *Z* denotes the concentration of PTE (mg/kg), and *z* is its ion valency. The percentage contribution (*X*) of each PTE was then calculated using the formula (Equation (8)):

(8) $$X=\left[\frac{\left(Z\;:\;z\right)\times 100}{Y}\right]$$

One-way analysis of variance (ANOVA), followed by Tukey’s Honest Significant Difference (HSD) test, was conducted to compare PTE concentrations across different plant tissues (statistical significance at *p* < 0.05). All statistical analyses and graphical visualization were performed using MS Excel 365 (Microsoft Corp, Redmond, WA, USA) and OriginPro 2024b (OriginLab, Northampton, MA, USA).

## 3. Results and Discussion

### 3.1. Water and Sediment Quality of Dal Lake

As depicted in Table 1, the water quality parameters of Dal Lake exhibit significant variations from permissible limits set by CPCB and BIS. The average pH of lake water was recorded to be alkaline (8.12) while sediment showed an acidic reaction (6.30). The observed pH difference between lake water and sediment can be attributed to several environmental and geochemical factors. The alkaline nature of lake water is likely influenced by high biological productivity, photosynthetic activity, and carbonate buffering, which increase hydroxyl ion concentration [46]. In contrast, the sediment exhibits an acidic reaction due to organic matter decomposition, microbial activity, and the accumulation of metal sulfides, which release protons and lower pH. Additionally, limited mixing between sediment and overlying water prevents pH equilibration, contributing to this disparity [47]. The BOD of 15.72 mg/L exceeds the permissible limit of 3 mg/L, indicating high organic pollution and microbial activity, likely due to anthropogenic inputs. Similarly, COD is high (135.15 mg/L), which is the result of a considerable load of non-biodegradable organic pollutants. TN and TP concentrations in water are also high. PTE concentrations in water, particularly Cd (0.06 mg/L), Cr (0.30 mg/L), and Ni (0.45 mg/L), exceeded regulatory limits. On the other hand, the bottom sediment data showed substantial accumulation of PTEs, with Fe (1610.51 mg/kg), Mn (31.48 mg/kg), and Cr (29.72 mg/kg) exceeding background levels. Herein, Cd concentration (0.94 mg/kg) exceeds the Canadian Sediment Quality Guidelines (CSQG) threshold (0.68 mg/kg) [48] and national sediment quality guidelines (SQGs) [49]. Moreover, DOM in sediments (455.37 mg/kg) further suggests high organic contamination. The observed EC in sediments (2.26 dS/m) was high compared to water (0.36 dS/m), which indicated ionic enrichment over time.

The observed water quality in Dal Lake indicated severe anthropogenic stress, primarily driven by unregulated sewage discharge, agricultural runoff, and industrial effluents [50]. The high BOD and COD levels indicate significant organic pollution, likely from untreated wastewater and decaying plant matter, which triggers microbial activity. Also, high nutrient levels, particularly TN and TP, indicated eutrophication possibilities [51], which could result in promoting algal blooms and oxygen depletion [52]. PTE contamination, particularly Cd, exceeding permissible limits, points to industrial and other commercial emissions, as well as leaching from urban waste. The acidic sediment pH and high EC also show that the lake has undergone long-term ionic enrichment and PTE accumulation, intensified by limited sediment removal. Previous studies on Dal Lake have shown significant natural and anthropogenic influences on its water and sediment chemistry. Water samples show a predominance of carbonate and silicate weathering, with lower pH and higher total dissolved solids in some areas due to sewage inputs [28]. PTE analysis also showed moderate enrichment of Cr, Ni, Cu, Zn, Pb, Fe, and Mn in sediments, with pollution levels increasing towards the central parts of the lake. Higher total organic carbon and nitrogen contents suggest eutrophic conditions in the lake basin [53]. In a study, Ahamad et al. [27] compared Dal and Nigeen lakes, showing higher levels of BOD, EC, COD, and PO_4_^3−^ in Dal Lake. Similarly, Kumar et al. [24] showed a significant decline in water quality over the past 40 years due to anthropogenic pressures, with decreased transparency and dissolved oxygen, and increased phosphates, nitrates, and chlorides.

### 3.2. PTE Concentrations in N. nucifera Tissues

As shown in Table 2, the distribution of PTEs in *N. nucifera* tissues varied significantly, with roots exhibiting the highest accumulation across most elements. Specifically, Cd was concentrated in roots (0.45 ± 0.05 mg/kg), significantly higher than in rhizomes (0.15 ± 0.03 mg/kg) and petioles (0.13 ± 0.02 mg/kg), while leaves and seeds contained the lowest concentrations. Similarly, Cu followed a similar trend, with roots accumulating the highest levels (9.50 ± 0.22 mg/kg), whereas seeds had the lowest (1.80 ± 0.05 mg/kg). Also, Cr and Co showed the highest accumulation in roots (3.60 ± 0.10 and 2.85 ± 0.08 mg/kg, respectively), with significant reductions in aerial parts. However, Fe was most abundant in roots (280.57 ± 18 mg/kg), followed by leaves (173.96 ± 8 mg/kg) and petioles (150.76 ± 10 mg/kg). Likewise, Mn and Ni concentrations were highest in roots, with significant declines in rhizomes, petioles, and leaves. Similarly, Zn exhibited the maximum accumulation in roots (40.30 ± 1.20 mg/kg), while seeds had the lowest levels (7.50 ± 0.25 mg/kg).

The uptake of PTEs by *N. nucifera* from lake water and sediment is affected by several factors such as root system, metal-binding capacity, and physiological adaptations. Roots act as primary sites for metal absorption due to their direct contact with sediment, where metals exist in bioavailable forms [54]. In particular, Cd is taken up due to its similarity to essential elements like Ca^2+^, allowing it to enter through Ca^2+^ transport channels [55]. Meanwhile, Cu is essential for enzymatic functions and is actively absorbed, but can accumulate excessively under contamination [56]. Similarly, Cr, particularly Cr(III), is absorbed due to its resemblance to essential micronutrients like Fe, while Co is taken up due to its role in nitrogen metabolism [57,58]. Fe is essential for chlorophyll synthesis, leading to high accumulation as evident in this study [59]. Also, Mn assists in photosynthesis and enzyme activation, Ni is required in trace amounts for urease activity, and Zn is absorbed as a cofactor for numerous enzymes [60]. Previously, Abd Rasid et al. [61] investigated the potential of *N. nucifera* in treating surface water and found that it can significantly reduce pollutant load while accumulating high levels of different mineral elements. Similarly, Liu et al. [62] also reported that *N. nucifera* could accumulate high levels of Cd in its tissues and clean the polluted waters. However, high Cd levels in plants could bring negative impacts on plants as well as consumers. In another study, Painuly et al. [63] also determined the As(III) accumulation capacity of *N. nucifera*. [64] found that *N. nucifera* has the potential to accumulate nine PTEs (Zn, Cu, Pb, Ni, Mn, Hg, Cr, Cd, and As) from irrigation lakes in Debarawewa and Galewela provinces in Sri Lanka.

### 3.3. Bioaccumulation and Translocation Factors

The BCF indicates the efficiency of *N. nucifera* in accumulating PTEs from its water body to its vegetative tissues (Table 3). In this, roots exhibited the highest BCF values for all PTEs, particularly Fe (95.11), Cr (12.00), and Mn (25.50), indicating their strong metal-binding capacity. Nevertheless, rhizomes also showed high accumulation, especially for Fe (40.70) and Zn (22.17). For aerial parts such as petioles and leaves, lower BCFs were recorded, exhibiting limited translocation from roots. Seeds consistently exhibited the lowest BCF values, which might be due to restricted PTE mobility. The high root BCF values showed the phytostabilization potential, where PTEs are sequestered in belowground tissues, minimizing translocation to edible parts. On the other hand, TF values (Table 4) showed the translocation efficiency of PTEs from roots to different tissues of *N. nucifera*. The highest TF was observed for Fe (0.62) from roots to leaves, which might be due to its active translocation via xylem transport for metabolic functions. Other PTEs exhibited lower translocation efficiencies, with Cd (0.09) showing the least mobility to leaves, suggesting strong retention in roots. Mn (0.67) and Cu (0.66) had relatively higher TF values from roots to rhizomes, indicating their moderate mobility. However, PTEs showed limited translocation to seeds (TF < 0.40). As given in Table 5, the results of ANE modeling showed a proportional contribution of each PTE in *N. nucifera* tissues. In this, Fe exhibited the highest accumulation across all tissues, accounting for 80.95–92.92% of total PTE uptake, indicating its requirement for plant metabolism and its strong affinity for root and shoot tissues. However, Zn also demonstrated a significant accumulation (6.10–9.79%), particularly in roots and rhizomes. Mn contributed 1.25–3.88%, while uptake of non-essential PTEs such as Cd, Cr, and Ni remained below 1.11%, which might be due to restricted translocation and possible detoxification mechanisms. In this, root tissues consistently accumulated the highest PTE concentrations, reinforcing their role as primary sites for PTE sequestration. The low participation of Cd (0.01–0.05%) indicates its minimal uptake, likely due to its toxicity. Overall, the results indicate that *N. nucifera* primarily stabilizes PTEs in below-ground tissues, minimizing translocation to aerial parts.

Several studies have investigated bioaccumulation and translocation factors in lake plants and modeled PTE accumulation. Galal and Farahat [65] examined *Pistia stratiotes* in Lake Mariut, finding bioaccumulation factors greater than one for most PTEs, except Cu, while translocation factors were also less than 1, suggesting its suitability for rhizofiltration. Bai et al. [66] evaluated the bioaccumulation potential of four aquatic plants in Taihu Lake, revealing that submerged plants, especially their stems, showed a closer relationship with PTEs in water and sediment compared to floating-leaf plants. Also, Skorbiłowicz et al. [67] used *Phragmites australis* as a bioindicator in the Bug River catchment, demonstrating that roots accumulated the highest levels of potentially toxic elements, making them necessary for monitoring PTE concentrations. In another study, Parzych et al. [45] applied ANE modeling to assess the capacity of *Salix viminalis* (willow) leaves and bark to accumulate PTEs from contaminated environments. They quantified the concentration of PTEs such as Cd, Pb, and Zn to assess the plant’s potential for phytoremediation and environmental monitoring.

### 3.4. Health Risk Assessment of PTEs

As shown in Table 6, the health risk assessment of PTE in *N. nucifera* edible tissues (rhizome and seeds only) was evaluated using HRI, DIM, and THQ indices. The results indicate that Cr exhibited the highest HRI in both rhizomes (1.8000) and seeds (0.5571), suggesting potential non-carcinogenic health risks upon prolonged consumption. Other PTEs, including Cd, Cu, and Fe, showed moderate HRI values, while Co, Mn, and Zn exhibited the lowest. The DIM values followed a similar trend, with Fe showing the highest intake, particularly in rhizomes (0.5143 mg/kg/day), while Cd had the lowest across both tissues. The THQ values for all PTEs remained below 1, indicating no immediate non-carcinogenic risk. The cumulative THQ (∑THQ) was 0.0054 for rhizomes and 0.0021 for seeds, further confirming low health risks. However, chronic exposure to Cr and Cd, given their relatively higher HRI, necessitates further monitoring. PTE contamination in edible *N. nucifera* tissues poses health risks to consumers.

The accumulation of PTEs in *N. nucifera* has ecological and health implications, as these elements can enter the food chain and affect aquatic ecosystems. Specifically, Cr is carcinogenic and induces oxidative stress, leading to cellular damage, which may impact aquatic organisms and plant health [68]. Plants primarily take up chromium in the hexavalent form [Cr(VI)] through root absorption, as it is more soluble and mobile in water compared to trivalent chromium [Cr(III)] [69]. However, Cr(III) can also be absorbed to a lesser extent if present in a bioavailable chelated form. In this, Cr(VI) is known to be more toxic than Cr(III) [70]. If contaminated *N. nucifera* is consumed by humans, Cd could accumulate in the kidneys and liver, causing renal dysfunction and bone demineralization, raising concerns about its bioaccumulation in edible plant parts [71]. While Cu is essential for enzymatic functions, excessive intake results in gastrointestinal distress and liver toxicity, posing risks to both plants and herbivorous consumers [72]. Fe is crucial for hemoglobin synthesis, but elevated levels contribute to oxidative stress and organ damage, potentially affecting plant physiology and aquatic biota [73]. Mn supports metabolism, yet excessive amounts impair neurological function, which may influence the health of organisms relying on contaminated water sources [74]. Ni exposure is linked to respiratory and cardiovascular issues, emphasizing risks for both aquatic and terrestrial ecosystems [75]. Co, essential for vitamin B12 synthesis, can be cardiotoxic at high concentrations, necessitating careful monitoring in sediment and water. Excess Zn disrupts immune function and nutrient absorption, potentially altering plant growth and ecosystem balance [76,77]. Understanding these effects is crucial for assessing environmental contamination, managing sediment quality, and ensuring the safe use of *N. nucifera* in food and medicine.

**Table 6:** Health risk assessment of PTEs in edible parts of *N. nucifera* plants grown in floating lake garden.

Element	HRI		DIM		THQ	
	Rhizome	Seeds	Rhizome	Seeds	Rhizome	Seeds
Cd	0.6429	0.3429	0.0006	0.0003	0.0006	0.0003
Cu	0.6696	0.1929	0.0268	0.0077	0.0007	0.0002
Cr	1.8000	0.5571	0.0090	0.0028	0.0018	0.0006
Co	0.3750	0.1071	0.0075	0.0021	0.0004	0.0001
Fe	0.7347	0.6429	0.5143	0.4500	0.0007	0.0006
Mn	0.2602	0.0643	0.0364	0.0090	0.0003	0.0001
Ni	0.5571	0.1607	0.0111	0.0032	0.0006	0.0002
Zn	0.3643	0.1071	0.1093	0.0321	0.0004	0.0001
∑THQ	-	-	-	-	0.0054	0.0021

Values are means followed by the standard deviation of 36 samples; Health Risk Index: HRI < 1 (safe), HRI ≥ 1 (potential risk); Daily Intake of Metal: Compared against tolerable daily intake limits of WHO/USEPA [78] (Cd: 1.00 μg/kg, Cu: 0.50 mg/kg, Cr: 25.00 μg/kg, Fe: 0.8 mg/kg, Mn: 0.06 mg/kg, Ni: 0.005 mg/kg, Zn: 1.00 mg/kg, Co: not defined); target hazard quotient: THQ < 1 (no risk), THQ ≥ 1 (potential health risk); ∑THQ (Cumulative Risk): ∑THQ < 1 (safe), ∑THQ ≥ 1 (potential non-carcinogenic risk).

Previously, Ologundudu et al. [79] assessed the PTE bioaccumulation potential of *Corchorus olitorius* (L.) and *Amaranthus hybridus* (L.) collected from a polluted dumpsite and found increased values of DIM and HRI. Obasi et al. [80] studied the health risks of PTEs (Hg, Cd, Mn, Pb, Cu, Co, As, Cr, Zn, and Mo) and found that Hg, Cd, and Pb had HRI and THQ > 1. In the Jammu and Kashmir region of India, Abou Fayssal et al. [35] studied the health risk of PTE uptake by saffron (*Crocus sativus* L.) cultivated in soils irrigated with domestic wastewater and Sarbal Lake water. They found that crops irrigated with domestic and Sarbal Lake water both had higher values of HRI, DIM, and THQ compared to those irrigated with borewell supplies. Therefore, these studies also corroborate the findings of the present study on the potential health risks associated with edible crop contamination grown in lakes.

## 4. Conclusions

The results of the present study indicated a significant accumulation of PTEs in *N. nucifera* cultivated in Dal Lake. The study revealed significant PTE contamination, with high sediment concentrations of Fe (1610.51 mg/kg), Mn (31.48 mg/kg), and Cr (29.72 mg/kg), preferential accumulation in roots (Fe uptake: 95.11), low translocation to edible parts, and Cr posing the highest non-carcinogenic risk (HRI = 1.8000 in rhizomes), while the cumulative THQ remained below 1, indicating no immediate risk but potential concerns with long-term exposure. In this, PTEs were primarily sequestered in root tissues, thereby reducing their translocation to edible parts. While the THQ values indicate no immediate health risk, the high HRI for Cr necessitates continued monitoring, especially given the chronic toxicity associated with PTE exposure. The findings are useful for policymakers and environmental agencies aiming to regulate lake water quality and mitigate contamination sources. However, this study has limitations, including the absence of long-term exposure assessments and potential variability in PTEs due to seasonal changes. Future research should focus on remediation strategies, such as phytoremediation enhancements, and evaluate the impact of varying environmental conditions on PTE bioaccumulation. Further studies are also needed to explore the genetic and physiological responses of *N. nucifera* to PTE stress, which may aid in the development of bioengineered aquatic plants for improved phytoremediation efficacy.

## Figures and Tables

**Table 1:** Water and bottom sediment quality of floating gardens in Dal Lake, Srinagar, India.

Parameter	Water			Bottom Sediment		
	Value	CPCB Standard	BIS Standard	Value	CSQG Standard	USEPA Standard
pH	8.12 ± 0.05	6.50–9.00	6.50–8.50	6.30 ± 0.04	-	6.00−8.50
EC (dS/m)	0.36 ± 0.02	0.75	0.75	2.26 ± 0.51	-	4.00
TDS	242.85 ± 6.95 mg/L	500.00	500.00	-	-	-
DOM	22.95 ± 6.20 mg/L	-	-	455.37 ± 79.05 mg/kg	-	0.10−5.00%
BOD	15.72 ± 0.42 mg/L	3.00	3.00	-	-	-
COD	135.15 ± 12.85 mg/L	250.00	250.00	-	-	-
TN	9.89 ± 1.28 mg/L	-	-	581.04 ± 45.09 mg/kg	-	0.02−0.50%
TP	5.60 ± 0.46 mg/L	-	-	94.20 ± 8.71 mg/kg	-	200−1000
Cd	0.06 ± 0.03 mg/L	0.01	0.01	0.94 ± 0.12 mg/kg	0.68	4.98
Cu	3.40 ± 0.29 mg/L	1.50	1.50	13.24 ± 1.87 mg/kg	18.70	149.00
Cr	0.30 ± 0.07 mg/L	0.05	0.05	29.72 ± 3.90 mg/kg	52.30	111.00
Co	0.52 ± 0.10 mg/L	-	-	11.63 ± 0.42 mg/kg	-	-
Fe	2.95 ± 0.16 mg/L	0.30	0.30	1610.51 ± 249.03 mg/kg	2.00–5.00%	-
Mn	0.50 ± 0.07 mg/L	0.10	0.10	31.48 ± 3.72 mg/kg	-	460.00
Ni	0.45 ± 0.09 mg/L	0.02	0.02	4.10 ± 0.21 mg/kg	15.90	48.60
Zn	1.15 ± 0.03 mg/L	5.00	5.00	66.86 ± 8.32 mg/kg	124.00	459.00

Values are means followed by the standard deviation of 36 samples; CPCB: Central Pollution Control Board, India; BIS: Bureau of Indian Standards; CSQG: Canadian Sediment Quality Guidelines; USEPA: United States Environmental Protection Agency.

**Table 2:** The concentration (mg/kg dwt.) of PTEs in different plant tissues of *N. nucifera* grown in the floating lake gardens of Dal Lake.

PTE	*N. nucifera* Tissues				
	Rhizome	Roots	Petiole	Leaves	Seeds
Cd	0.15 ± 0.03 b	0.45 ± 0.05 a	0.13 ± 0.02 b	0.04 ± 0.01 c	0.08 ± 0.01 c
Cu	6.25 ± 0.15 b	9.50 ± 0.22 a	4.80 ± 0.10 c	3.20 ± 0.08 d	1.80 ± 0.05 e
Cr	2.10 ± 0.07 b	3.60 ± 0.10 a	1.75 ± 0.05 c	1.10 ± 0.03 d	0.65 ± 0.02 e
Co	1.75 ± 0.06 b	2.85 ± 0.08 a	1.30 ± 0.04 c	0.90 ± 0.02 d	0.50 ± 0.01 e
Fe	120.06 ± 12 c	280.57 ± 18 a	150.76 ± 10 b	173.96 ± 8 b	105.40 ± 5 c
Mn	8.50 ± 0.20 b	12.75 ± 0.35 a	6.40 ± 0.15 c	4.25 ± 0.10 d	2.10 ± 0.05 e
Ni	2.60 ± 0.08 b	4.30 ± 0.12 a	2.00 ± 0.06 c	1.40 ± 0.04 d	0.75 ± 0.02 e
Zn	25.50 ± 0.80 b	40.30 ± 1.20 a	18.40 ± 0.55 c	12.60 ± 0.40 d	7.50 ± 0.25 e

Values are means followed by the standard deviation of 36 samples; different letters within each row indicate significant differences among plant tissues (*p* < 0.05) based on Tukey’s HSD.

**Table 3:** Bioconcentration factors (BCFs) of PTE uptake by *N. nucifera* plant tissues.

BCF	*N. nucifera* Tissues				
	Rhizome	Roots	Petiole	Leaves	Seeds
Cd	2.50	7.50	2.17	0.67	1.33
Cu	1.84	2.79	1.41	0.94	0.53
Cr	7.00	12.00	5.83	3.67	2.17
Co	3.37	5.48	2.50	1.73	0.96
Fe	40.70	95.11	51.11	58.97	35.73
Mn	17.00	25.50	12.80	8.50	4.20
Ni	5.78	9.56	4.44	3.11	1.67
Zn	22.17	35.04	16.00	10.96	6.52

Values are means followed by the standard deviation of 36 samples.

**Table 4:** Translocation factors (TFs) of PTE uptake by *N. nucifera* plant tissues.

TF	*N. nucifera* Tissues			
	Root → Rhizome	Root → Petiole	Root → Leaves	Root → Seed
Cd	0.33	0.29	0.09	0.18
Cu	0.66	0.51	0.34	0.19
Cr	0.58	0.49	0.31	0.18
Co	0.61	0.46	0.32	0.18
Fe	0.43	0.54	0.62	0.38
Mn	0.67	0.50	0.33	0.16
Ni	0.60	0.47	0.33	0.17
Zn	0.63	0.46	0.31	0.19

Values are means followed by the standard deviation of 36 samples.

**Table 5:** Accumulation of nutrient elements (ANEs) modeling for PTE uptake by *N. nucifera* plant tissues.

Element	*N. nucifera* Tissues									
	Rhizome		Roots		Petiole		Leaves		Seeds	
	*X*	*P* (%)	*X*	*P* (%)	*X*	*P* (%)	*X*	*P* (%)	*X*	*P* (%)
∑PTE (mmol_c_/Kg)	796.73	100.00	1745.79	100.00	922.77	100.00	1010.73	100.00	609.36	100.00
Cd	0.27	0.03	0.80	0.05	0.23	0.03	0.07	0.01	0.14	0.02
Cu	19.67	2.47	29.90	1.71	15.11	1.64	10.07	1.00	5.67	0.93
Cr	8.08	1.01	13.85	0.79	6.73	0.73	4.23	0.42	2.50	0.41
Co	5.94	0.75	9.67	0.55	4.41	0.48	3.05	0.30	1.70	0.28
Fe	644.96	80.95	1507.23	86.33	809.88	87.77	934.52	92.46	566.21	92.92
Mn	30.94	3.88	46.42	2.66	23.30	2.52	15.47	1.53	7.64	1.25
Ni	8.86	1.11	14.65	0.84	6.82	0.74	4.77	0.47	2.56	0.42
Zn	78.01	9.79	123.28	7.06	56.29	6.10	38.54	3.81	22.94	3.77

Values are means followed by the standard deviation of 36 samples; *X*: total accumulation of the respective PTEs in *N. nucifera* tissues, expressed in mmol_c_/kg; *P* (%): percentage contribution of the respective PTE to the total PTE accumulation in each tissue. WHO permissible limits in vegetables: Cd: 0.02, Cu: 10.00, Cr: 1.30, Ni: 10.00, Zn: 0.60, Co, Fe, and Mn: not defined.

## Data Availability

The original contributions presented in this study are included in the article. Further inquiries can be directed to the corresponding author.
